# The Prothrombotic Tendency in Metabolic Syndrome: Focus on the Potential Mechanisms Involved in Impaired Haemostasis and Fibrinolytic Balance

**DOI:** 10.6064/2012/525374

**Published:** 2012-08-30

**Authors:** Isabella Russo

**Affiliations:** Internal Medicine and Metabolic Disease Unit, Department of Clinical and Biological Sciences of the Turin University, San Luigi Gonzaga Hospital, 10043 Orbassano, Italy

## Abstract

The metabolic syndrome is a clinical disorder characterized by impairment of glucose metabolism, increased arterial blood pressure, and abdominal obesity. The presence of these clinical features exposes patients to a high risk of atherothrombotic cardiovascular events. The pathogenesis of atherothrombosis in the metabolic syndrome is multifactorial, requiring a close relationship among the main components of the metabolic syndrome, including insulin resistance, alterations of glycaemic and lipid pattern, haemodynamic impairment, and early appearance of endothelial dysfunction. Furthermore, haemostatic alterations involving coagulation balance, fibrinolysis, and platelet function play a relevant role both in the progression of the arterial wall damage and in acute vascular events. The mechanisms linking abdominal obesity with prothrombotic changes in the metabolic syndrome have been identified and partially elucidated on the basis of alterations of each haemostatic variable and defined through the evidence of peculiar dysfunctions in the endocrine activity of adipose tissue responsible of vascular impairment, prothrombotic tendency, and low-grade chronic inflammation. This paper will focus on the direct role of adipose tissue on prothrombotic tendency in patients affected by metabolic syndrome, with adipocytes being able to produce and/or release cytokines and adipokines which deeply influence haemostatic/fibrinolytic balance, platelet function, and proinflammatory state.

## 1. Introduction

### 1.1. General Background

The Framingham Heart Study was one of the first epidemiological studies which showed the causal relationship between obesity and cardiovascular disease [1–3]. Furthermore, a widespread literature, including the relevant Kuopio Ischaemic Heart Disease Risk Factor Study, a population-based, prospective cohort study of 1,209 Finnish men, supported the conclusion that not only the excess of adipose tissue, but also the distribution of adiposity is essential feature influencing the cardiovascular risk [2]. In particular, central obesity (fat in the trunk and/or abdomen)—which characterizes the metabolic syndrome [3, 4]—confers a higher degree of cardiovascular risk than peripheral adiposity, being closely associated with an elevated risk of cardiovascular morbidity and mortality due to atherothrombotic events [2–8]. The recent INTERHEART study, a standardized case-control study to evaluate the association of risk factors of myocardial infarction in 27,098 participants in 52 countries, confirmed this finding: actually, this study showed that the waist-to-hip ratio (WHR)—a reliable index of central obesity—was the strongest anthropometric predictor of myocardial infarction both in men and women across all investigated ages and ethnic groups, whereas the association of body mass index (BMI) with cardiovascular events was not evident, when adjusted for the other risk factors [9, 10]. Also in the INTERSTROKE study, an international, multicenter case-control study which investigated patients (*n* = 6,000) with ischaemic or haemorrhagic stroke within 72 hours of hospital admission and in whom CT (computed tomography) or MRI (magnetic resonance imaging) was globally performed [11], the WHR was a strong anthropometric predictor of stroke across all investigated ethnic groups. As widely reviewed by several authors, the increase in risk of stroke in subjects with the metabolic syndrome, compared with individuals without this disorder, is a 3-fold increase [12, 13] 

### 1.2. Definition of the Metabolic Syndrome

The metabolic syndrome is a complex disorder characterized by the presence of a clustering of metabolic risk factors usually in a single individual associated with the presence of central obesity and a strong association with diabetes and cardiovascular disease morbidity and mortality.

According to the National Cholesterol Education Program (NCEP)'s Adult Treatment Panel III criteria [14], the diagnosis of metabolic syndrome requires the copresence of at least three of the following: (i) central obesity characterized by waist circumference >88 cm in women and >102 cm in men, (ii) fasting blood glucose ≥6.1 mmol/L or currently using hypoglycemic drugs, (iii) fasting blood triglycerides (TG) ≥1.69 mmol/L or currently using specific drug treatment, (iv) fasting blood cholesterol HDL <1.03 mmol/L in men and <1.29 mmol/L in women, (v) systolic blood pressure ≥130 mmHg, diastolic blood pressure ≥85 mmHg, or currently using antihypertensive drugs. In a revised version of NCEP by the American Heart Association and National Heart Lung Blood Institute, cut-off value of fasting blood glucose is ≥5.6 mmol/L [15].

According to the International Diabetes Federation (IDF), the diagnosis of metabolic syndrome requires evidence of central obesity with cutoff values of waist circumference depending on gender and ethnic group origin (for instance, cut-off is ≥94 cm for Europid males and ≥80 cm for Europid females) together with the other risk factors as defined in revised NCEP [16].

 A Joint Interim Statement (JIS) by many medical organizations confirmed that waist cut-off values defining high-risk groups are different between populations but criticised the criteria of abdominal obesity measurement as a prerequisite of metabolic syndrome [17].

Thus, at present, a unifying definition is lacking [18], but it is well established that the metabolic syndrome is a constellation of disorders increasing the risk of cardiovascular diseases [19] and type II diabetes [20, 21].

### 1.3. Epidemiology

Due to the absence of an unifying definition, the metabolic syndrome can be present in several forms according to the combination of the different components; therefore, the exact evaluation of prevalence of the metabolic syndrome is quite different both in the United States and in Europe. Recent data using the World Health Organization (WHO) definition and data from National Health and Nutrition Examination Survey III (NHANES III) indicated that the age-adjusted prevalence of the metabolic syndrome is estimated at 23.7% and increases with the increasing prevalence of central obesity which affects more than 40% of subjects older than 60 years in the United States. Based on the data from 2000, 47 millions of individuals in the United States have the metabolic syndrome [22–26], indicating that about one quarter of America's population has the metabolic syndrome, and about 84% of them present abdominal obesity on the basis of the criteria indicated by NCEP ATPIII.

As mentioned, the metabolic syndrome is recognized as a pervasive condition related to cardiovascular atherosclerotic ischemic disease due to the presence of a clustering of three or more risk factors, including abdominal obesity, atherogenic dyslipidaemia (hypertriglyceridaemia, low HDL cholesterol), raised blood pressure, insulin resistance with or without impaired glucose tolerance, and proinflammatory state [18, 27]. Considering the epidemiologic data, the presence of previously mentioned risk factors increases the 16-year risk of coronary heart disease (angioplasty, unstable angina, myocardial infarction, and coronary death) of 2.39 times in men and 5.9 times in women [28].

**Figure 1:**
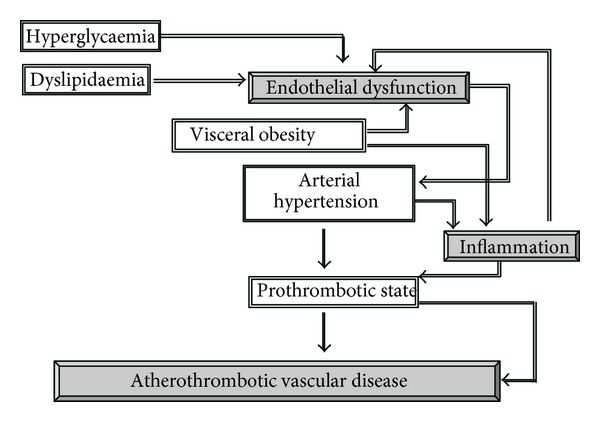
Potential mechanisms linking visceral obesity, which characterizes the metabolic syndrome, inflammation, and atherothrombotic vascular disease.

The metabolic syndrome is clearly related to central obesity and is associated with the development of atherosclerotic vascular damage and an increased susceptibility to the clinical manifestations of atherothrombosis owing to the presence of a network of pathogenic factors (Figure 1), including chronic low-grade inflammatory state, impaired glucose metabolism, atherogenic dyslipidaemia, arterial hypertension, endothelial dysfunction, and prothrombotic alterations [7, 8, 29, 30]. The insulin-resistance state plays an important pathophysiologic role even in nondiabetic individuals [31] representing a significant link among components of metabolic syndrome even if not necessarily all subjects affected by metabolic syndrome are insulin resistant [32]

This constellation of disorders indicates that insulin resistance and type 2 diabetes mellitus are a major cause of cardiovascular disease, with consequent disabilities and mortality on a global scale, representing also a relevant cause of financial cost in the different healthcare systems. 

**Figure 2:**
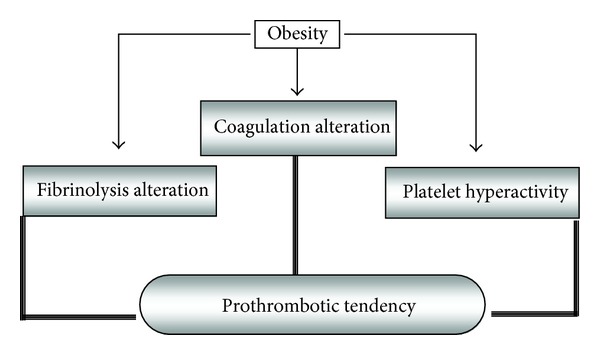
Prothrombotic disorders in obesity.

Apart from metabolic and haemodynamic alterations, central obesity is characterized by an evident prothrombotic tendency (Figure 2) with increased thrombin generation and platelet activation and decreased fibrinolysis [29, 30, 33, 34], which contribute both to atherogenesis and acute atherothrombotic events via increased vascular deposition of platelets and fibrinous products. 

In this paper, the main alterations responsible of impairment of the haemostatic balance in patients with the metabolic syndrome will be firstly taken into account, considering their relationship with hormonal and metabolic alterations related to the increased omental adipose tissue (central obesity) and their relevance in the increased prevalence of cardiovascular events. Obesity is a pivotal risk factor for coronary heart disease, ventricular dysfunction, congestive heart failure, and cardiac arrhythmias [35, 36] and could explain the epidemiological observation which describes an increased prevalence of heart failure in the metabolic syndrome [37].

## 2. Alterations of the Coagulation Cascade in the Metabolic Syndrome

### 2.1. Haemostatic Balance in Physiological and Pathophysiological Conditions

Haemostasis is a process initiated when a damage occurs to the wall of a blood vessel and culminates with the formation of a stable clot. This occurs in three stages: vasoconstriction, platelet response (white clot formation), and blood coagulation; the last process depends on the tight balancing between the activation of coagulation and fibrinolytic systems activated for the repair of the blood vessel and determining clot dissolution and restoration of the vessel function [38–40]. The clot formation depends on two pathways: the slower intrinsic one is linked to circulating coagulation factors, such as factors IXa and VIIIa; the more rapid extrinsic one is activated when blood is exposed to an extravascular factor such as tissue factor (TF) [38–40]. Factor VII (FVII) plays a key role in the initiation of extrinsic pathway when it forms a complex with TF exposed and released by damaged or activated endothelium or by disrupted atheromatous plaque [40]. Activation of the coagulation system induces the conversion of prothrombin in thrombin, which determines the conversion of fibrinogen into fibrin and induces platelet activation [38, 39].

Alterations of the coagulation cascade and/or fibrinolysis, especially in the presence of a low-grade inflammation, are key pathogenic components of the atherothrombotic process which underlies acute coronary and cerebrovascular events [40]. In these conditions, the activation of inflammatory mechanisms is strictly dependent on interaction among different cell types, such as platelets, circulating and resident leukocytes, and cells of the vascular wall, such as endothelial and vascular smooth muscle cells (VSMCs) [41]. 

Recently, the release of cell fragments known as microvesicles or microparticles (MPs) has been recognized as an integral part of the thrombotic process [42, 43].

 MPs are small, irregularly shaped, phospholipid vesicles (200–1,000 nm) containing procoagulant and proinflammatory mediators, released in blood as a consequence of activation or apoptosis of endothelial cells, leukocytes, and platelets [44–46]. They express membrane antigens that reflect their cellular origin, as well as a broad array of other proteins inherent to their parental cells (e.g., MPs derived from white blood cells contain TF which is able to activate extrinsic coagulation cascade). 

MPs have been recognized in blood from healthy individuals, but their number is markedly increased in patients under pathological states, such as disseminated intravascular coagulation, diabetes, immune-mediated thrombosis, acute coronary syndromes, systemic inflammatory diseases, and metabolic syndrome [42, 47], allowing to hypothesize a role as vectors of biological messages, such as induction of endothelial and vascular dysfunction or platelet activation [45, 47].

### 2.2. Hemostatic Alterations Identified in the Metabolic Syndrome

The impairment of hemostatic balance identified in subjects with central obesity includes alterations of both intrinsic and extrinsic pathways with increased levels of factor VIII (FVIII) and von Willebrand factor (vWF), TF, FVII, and fibrinogen [33, 48]. 

#### 2.2.1. Von Willebrand Factor (vWF) and Factor VIII (FVIII)

vWF is a multimeric glycoprotein synthesized and secreted by vascular endothelial cells and megakaryocytes [49]; it promotes platelet adhesion to the vascular subendothelium exposed following endothelial cell damage and is involved in platelet aggregation [49]. vWF is required also for stability of FVIII, and the two proteins circulate as a complex and cooperate both in platelet adhesion and in clot formation; therefore, the levels of vWF and FVIII are closely associated [50]. Like fibrinogen, both vWF and FVIII are acute-phase proteins.

An elevated circulating level of vWF is a marker of endothelial cell damage [48] and, together with other cardiovascular risk factors such as tissue plasminogen activator (t-PA) and plasminogen activator inhibitor (PAI-1), predicts the risk of future cardiovascular events in patients with vascular disorders such as angina [48, 51].

In the Atherosclerosis Risk in Communities (ARIC) study, both vWF and FVIII were associated with components of the metabolic syndrome including BMI, plasma insulin levels, and triglyceridemia [50]. It has been hypothesized that the mechanisms linking elevated levels of vWF/FVIII complex to insulin resistance and central obesity are related to the presence of underlying endothelial dysfunction [52] and/or proinflammatory milieu [53] even though this association is still controversial [54].

#### 2.2.2. Tissue Factor (TF)

TF is the primary *in vivo* initiator of the extrinsic coagulation cascade; it functions as a transmembrane receptor for factor VII/factor VII activated (FVII/VIIa) and as a signal molecule able to increase the transcription of genes involved in inflammation, apoptosis, embryonic development, and cell migration [55].

 The complex TF-FVIIa catalyzes the conversion of factor IX (FIX) and factor X (FX) into their activated forms, serving as the main cofactor to lead to fibrin formation both in physiological and pathological conditions [55, 56]. 

In physiological conditions, TF expression is confined only in cells of the adventitia layer which surrounds blood vessels, forming an envelope that prevents blood extravasation; in pathological states, however, TF is expressed also by activated endothelium and monocytes [55, 57], macrophage-derived foam cells, and VSMC [56]. TF is recognized also in the lipid core of unstable atherosclerotic lesions, allowing to hypothesize a relevant role in the pathogenesis of atherothrombotic events [56, 57]. Circulating TF has been identified also within MPs derived from activated and apoptotic cells [71].

Under stimulation of the proinflammatory cytokine transforming growth factor-*β* (TGF-*β*), adipocytes synthesize TF in a small amount [58], and increased circulating levels of TF have been identified as a relevant factor involved in prothrombotic tendency of patients affected by high-grade obesity [59]. Furthermore, leptin influences TF expression in human peripheral blood mononuclear cells [60, 61]. 

 An increased number of circulating MPs containing TF have been detected in patients with central obesity with a positive relationship with components of the metabolic syndrome [62].

#### 2.2.3. Factor VII (FVII)

FVII is a 50 kDa vitamin K-dependent serine protease synthesized in the liver which plays a pivotal role in the activation of the extrinsic coagulation cascade together with TF [55, 56]. 

An association between FVII levels and coronary events has been shown in several but not all studies [48, 63]. In the Northwick Park Heart Study (NPHS), the levels of FVII coagulant activity (FVII:c) were associated with fatal and not fatal coronary artery disease [64].

Circulating FVII:c may bind to triglyceride-rich lipoproteins, and its plasma levels are related to those of chylomicron and very-low density lipoprotein (VLDL) fractions [65]; deficient postprandial catabolism of these lipoproteins may prolong FVII half-life and increase its plasma concentrations [66].

#### 2.2.4. Fibrinogen

Fibrinogen is a heterodimer composed of three pairs of nonidentical polypeptide chains (A*α*, B*β*, and *γ*) synthesized by the liver [38, 39]. Its plasma levels influence thrombogenesis and affect the rheology of blood flow and the platelet aggregation [67]; furthermore, it acts as an acute-phase reactant produced under the stimulation of interleukin-6 (IL-6) [67]. 

Prospective epidemiological studies in general population constantly found an association between raised plasma levels of fibrinogen and increased risk of cardiovascular events, inducing to consider fibrinogen a strong and independent atherothrombotic risk factor through its effects on blood viscosity, coagulation, platelet function, and inflammation [67, 68].

Although the relationship between fibrinogen and features of the insulin resistance syndrome is weaker than for other hemostatic factors such as PAI-1 and FVII [33, 48, 69, 70], epidemiological studies have consistently found a significant association between levels of fibrinogen and those of insulin [33]; on this basis, several studies enclosed the increase of fibrinogen in the cluster of cardiovascular risk factors of the metabolic syndrome [67, 69, 70].

In particular, elevated fibrinogen levels could be explained by the proinflammatory state of central obesity and insulin resistance states, characterized by elevated synthesis and secretion of IL-6 and other proinflammatory cytokines.

#### 2.2.5. The Circulating Microparticles (MPs)

As previously reviewed, circulating MPs can contain coagulation factors—TF, in particular—and contribute to the amplification of the thrombotic response [62, 71–74].

Several lines of evidence indicate that patients with central obesity, as well as with diabetes mellitus, have increased circulating levels of MPs compared with healthy subjects [72, 75], including MPs derived from platelet, endothelial cells, erythrocytes, and white blood cells [75].

Furthermore, *in vitro* exposure of cultured endothelial cells to MPs derived from patients with the metabolic syndrome reduced both nitric oxide (NO) and superoxide anion (O_2_^−^) production with a reduction of endothelial-type constitutive NO synthase (ecNOS) activity [76]. 

## 3. Alterations of Fibrinolysis in Central Obesity

### 3.1. The Fibrinolytic Pathway in Physiological and Pathophysiological Conditions

The fibrinolytic pathway is responsible for the removal of fibrin from the circulation through its degradation within the thrombus; therefore, it plays a pivotal role in disintegrating clots and maintaining vascular patency [77].

Fibrinolysis is activated by the enzymatic conversion of the proenzyme plasminogen into the active enzyme plasmin which degrades fibrin into soluble fibrin degradation products (FDPs) [77, 78]; this process is mediated by tissue type (t-PA) and urokinase-type (u-PA) plasminogen activators [77, 78]. 

Plasma fibrinolytic activity is tightly regulated by inhibitors, mainly represented by *α*2-antiplasmin (*α*2-AP) at the level of plasmin and by plasminogen activator inhibitors (PAI-1 and PAI-2) at the level of the plasminogen activators [78]. Furthermore, a further inhibitory mechanism of fibrinolysis dependent on the presence of thrombin-activatable fibrinolysis inhibitor (TAFI) has been identified [79].

*α*2-AP is the primary physiological inhibitor of plasmin which is rapidly inhibited in plasma but partly protected from *α*2-AP action when it is bound to fibrin [77].

The primary inhibitor of the fibrinolytic system is PAI-1—a single-chain glycoprotein (379 to 381 amino acids; MW: 48 kDa), member of the superfamily of the serine protease inhibitors—which inhibits plasminogen activation by binding with tPA to form the PAI-1/tPA complex [78].

Liver, platelets, endothelial cells, and VSMC are the main sources of PAI-1, but other cell types can synthesize and secrete this protein [78].

TAFI—known as plasma procarboxypeptidases B, R, and U, EC 3.4.17.20—is synthesized in the liver and secreted as a propeptide consisting of 401 amino acids, with a molecular weight of 60 kDa; it is present also in platelets and endothelial cells [80–82]. The zymogen is activated through a proteolytic cleavage by thrombin, thrombin/thrombomodulin, and plasmin, and in activated form (TAFIa), it attenuates fibrinolysis by protecting the lysis of the fibrin clot through the remotion of C-terminal lysine residues which act as anchoring sites for tPA and plasminogen [83].

Hypofibrinolysis, that facilitates fibrin deposition in vessel wall, is deeply involved in the increase of atherothrombotic events, especially if associated to a prothrombotic tendency related to high plasma concentrations of vWF/FVIII complex, TF, FVII, and fibrinogen [64, 84–88]. In particular, a reduced fibrinolysis due to high levels of circulating PAI-1 predicts cardiovascular events in young men after myocardial infarction [86, 89] and in patients with angina pectoris [86].

### 3.2. Alterations of the Fibrinolytic System Identified in Central Obesity

#### 3.2.1. Plasminogen Activator Inhibitor (PAI-1)

**Figure 3:**
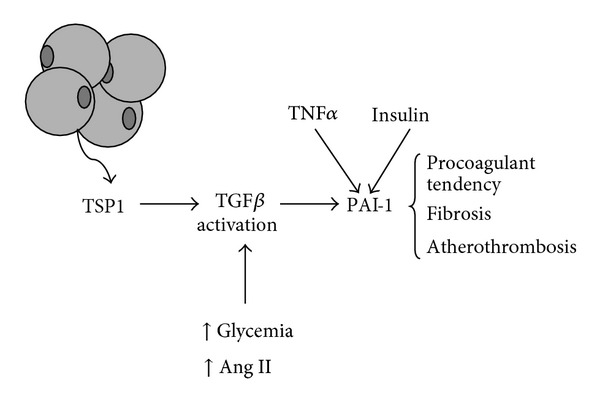
Control of PAI-1 synthesis and release in adipose tissue. It is underlined the role of thrombospondin-1 (TSP1), an adipokine which activates transforming growth factor-*β* (TGF-*β*), and consequently PAI-1 synthesis and release in adipose tissue. TGF-*β* is also activated by high glucose and angiotensin II (Ang II). The synthesis of PAI-1, which exerts prothrombotic and proatherogenic actions, is increased also by TNF-*α* and insulin.

Adipocytes synthesize PAI-1 through a mechanism regulated by insulin, glucocorticoids, angiotensin II, and cytokines (Figure 3). The proinflammatory cytokines tumor necrosis factor-*α* (TNF-*α*) and TGF-*β*, in particular play a relevant role in PAI-1 oversecretion from the adipose tissue [90, 91]. Also hyperinsulinemia and alterations of circulating lipid pattern characterizing central obesity—such as increased circulating levels of free fatty acids or VLDL lipoproteins—have been shown to stimulate PAI-1 production by hepatocytes [92].

The metabolic syndrome is usually characterized by elevated circulating level of PAI-1 [33, 48, 93, 94], and some Authors established a direct relationship between PAI-1 levels and visceral adipose tissue mass. 

There are lines of evidence that a major role in the elevation of PAI-1 in obesity is attributable to upregulated production by adipose tissue itself [95, 96], and, recently, it has been shown that a systemic inflammation induces a significant increase of gene expression of PAI-1 in adipose tissue followed by increase of PAI-1 levels in plasma [97].

 The alteration of the fibrinolytic system related to increased circulating levels of PAI-1 is considered to have a relevant role in the prothrombotic tendency associated with obesity [96].

A derangement of the endogenous fibrinolytic system could justify, at least in part, clinical observations which evidenced a resistance to intravenous thrombolysis in acute middle cerebral artery ischemic stroke in women with the metabolic syndrome [98].

Elevated levels of PAI-1 have been also recognized among type 2 diabetic patients [99] and predict myocardial infarction and stroke [86, 87, 100]. 

#### 3.2.2. Thrombin-Activatable Fibrinolysis Inhibitor (TAFI)

Studies showed increased circulating levels of TAFI antigen and TAFI activity in obese patients [80, 101]. Clear evidence of adipocyte-dependent TAFI synthesis is not available [101, 102]; however, several studies showed that the hormonal alterations related to central obesity influence the synthesis of this antifibrinolytic factor by other cell types: actually, insulin regulates TAFI gene expression in hepatocytes [102], and proinflammatory cytokines increase its production, inducing to consider this enzyme as an acute-phase reactant [103, 104].

## 4. Alterations of the Platelet Function in the Metabolic Syndrome

### 4.1. Role of Platelets in the Atherothrombosis

As extensively reviewed [41, 105], the formation of platelet plug plays a pivotal role not only in haemostasis, but also in vascular inflammation and atherothrombosis by adhering to activated endothelial cells or to the subendothelium layer in the presence of vascular damage, by releasing storage granules, and by aggregating to form thrombi. Activation of platelets is triggered by adhesion molecules present in the subendothelial matrix components, such as collagen, vWF, and fibronectin and is accompanied by the increased exposure of platelet membrane glycoprotein receptors able to bind fibrinogen and other proteins. Platelet activation and formation of aggregates are triggered also by thrombin and endogenous mediators released from storage granules including adenosine 5-diphosphate (ADP), platelet activating factor (PAF), and thromboxane A_2_ (TXA_2_). The adhesion/aggregation is regulated by the balance between proaggregants and circulating antiaggregants which are mainly represented by endothelium-derived prostacyclin (PGI_2_) and nitric oxide (NO).

### 4.2. Alterations of Platelet Function in Central Obesity

Several defects of platelet function have been identified in insulin-resistant states and central obesity, as recently reviewed [34, 106]. 

Mean platelet volume, a parameter directly related to *in vivo* platelet activation [107], has been described as increased in patients with obesity [108] and metabolic syndrome [109] and, as recently shown, does not differ in obese patients in association with metabolic syndrome [110].

Furthermore, another index of *in vivo* platelet activation—that is urinary excretion of 11-dehydro-TXB_2_, the major enzymatic metabolite of TXA_2_—is increased in women affected by visceral obesity, compared to nonobese women [111]. Surprisingly, it has been recently shown that the serum levels of TXB2, the circulating stable metabolite of TXA2, are significantly lower in insulin-sensitive morbidly obese subjects than in obese and lean subjects, suggesting that a reduction of platelet activation could play a role in the paradoxical protection of the morbidly obese subjects from coronary atherosclerosis, despite higher circulating levels of leptin and C-reactive protein [112].

Overweight and obese insulin-resistant subjects exhibited also enhanced plasma concentrations of P-selectin—a marker of platelet activation exposed in cell surface and released in circulating blood—in comparison with controls [113].

The prevalence of activated platelets is usually associated to the increase of other prothrombotic proteins; in particular, some authors found, in patients with severe obesity and insulin resistance, increased levels of soluble CD40 ligand (sCD40L) [114–116], prothrombin fragment F1+2 [114–116], and platelet-derived microparticles (PMPs) [117].

CD40 ligand (CD40L) is a trimeric transmembrane protein structurally related to TNF-*α* superfamily, with a dual prothrombotic and proinflammatory role [116]. 

CD40L is stored in the cytoplasm of resting platelets and rapidly exposed on cell surface after activation [116]. The surface-exposed fraction is subsequently cleaved with the release of a soluble fragment sCD40L which exerts prothrombotic effects by increasing the stability of newly formed platelet aggregates; furthermore, sCD40L activates inflammatory responses in cells of the vascular wall, including production of reactive oxygen species (ROS) by endothelium, enhanced expression of adhesion molecules: vascular cell adhesion molecule-1 (VCAM-1), intercellular adhesion molecule-1 (ICAM-1), and E-selectin by endothelial and VSMC, and increased secretion of cytokines and chemokines [116].

PMPs with a diameter less than 0.1 micron are identified by the presence of glycoprotein CD42b and CD42a [117]; they are released by platelets following agonist-induced activation or high shear stress [117], and contain proinflammatory and prothrombinase activities [118, 119]. 

Recent finding showed that circulating levels of PMPs are elevated in obese nondiabetic subjects in comparison with nonobese controls with a positive correlation with BMI and waist circumference: this fact is related to enhanced *in vivo *platelet activation [119, 120]. Furthermore, it has been found an effect of weight loss on PMP overproduction [120, 121].

As it will be extensively described in the next part of the paper, several available studies identified as main defect of the platelet function in subjects with central obesity a decreased sensitivity to mediators playing a physiological role in the reduction of platelet sensitivity to proaggregating stimuli, including insulin, NO, and cyclic nucleotides themselves [34, 122–124]. 

#### 4.2.1. Role of Insulin and Insulin Resistance in the Modulation of Platelet Function

Membrane of human platelets expresses insulin receptors, with a density similar to that present in cell types responsive to the metabolic actions of the hormone [125]. Platelet insulin receptors activate the classical intracellular pathway of insulin signaling, although without the increase of glucose uptake [125]. 

In insulin-sensitive subjects, the hormone exerts an antiaggregating activity recognized by both *in vitro* [126–128] and *in vivo* studies through euglycemic hyperinsulinemic clamps [126, 127, 129]. Furthermore, insulin decreases intraplatelet concentrations of calcium and prevents the angiotensin II-stimulated transient calcium increase in cytosol [130]. 

*In vivo* studies showed also that insulin infusion in euglycemic conditions decreases the platelet deposition to collagen in flowing whole blood perfusion, impairs primary hemostasis under high shear rate conditions, and uniformly inhibits platelet aggregation in response to multiple agonists [131]. 

The insulin effects are, at least in part, dependent on the activation of ecNOS through an increase of intraplatelet 3′,5′-cyclic guanosine monophosphate (cGMP) mediated by the stimulation of soluble guanylate cyclase activity [132]; through this mechanism, insulin elicits also a rapid increase of intraplatelet concentrations of 3′,5′-cyclic adenosine monophosphate (cAMP) via cGMP-dependent inhibition of a cAMP phosphodiesterase [132].

It is known that cGMP and cAMP, acting predominantly via specific protein kinases, block several steps of the agonist-induced elevation of cytosolic calcium, a basic mechanism of platelet activation [133–135], thus inhibiting platelet function. 

Some *in vitro* studies reported that insulin interferes also with activation of the purinergic receptor P_2_Y_12_, which mediates the ADP-induced platelet activation [136].

In conditions of insulin resistance, such as central obesity, type 2 diabetes mellitus with obesity, and essential arterial hypertension, a deep reduction of platelet sensitivity to the antiaggregating effects of insulin has been reported [34, 137–139]. 

Also the effects of insulin infusion on platelet deposition to collagen in flowing whole blood perfusion are lost in obese insulin-resistant subjects [131]. 

#### 4.2.2. Defective Sensitivity to Other Antiaggregating Agents

Studies showed that platelets from obese subjects and obese type 2 diabetic patients and individual with the metabolic syndrome are resistant to the antiaggregating effects of NO donors, including glyceryl trinitrate (GTN) and sodium nitroprusside (SNP) [140, 141]. The decreased sensitivity to the antiaggregating effects of GTN and SNP was associated with reduced intraplatelet accumulation of cGMP [140, 141]. 

This impairment of platelet response to NO (defined also as “NO resistance”) is similar to that identified by other authors in platelets of nondiabetic patients affected by coronary ischemic disease [142–146].

Obese subjects are also resistant to the antiaggregating effects of both PGI_2_ and adenosine, which act through the adenylate cyclase/cAMP pathway [141]. In particular, the ability of PGI_2_ to increase cAMP is impaired in visceral obesity [141].

In addition, it was observed that platelets from obese subjects are resistant to the antiaggregating effects of the cyclic nucleotides themselves, as evidenced by experiments with cell permeable analogues of both cGMP and cAMP [141], suggesting the presence of abnormalities in intraplatelet calcium fluxes handling, because both cGMP and cAMP exert their effects mainly through a reduction of intracellular calcium [135]. Furthermore, the same authors observed that platelets from subjects affected by central obesity show a reduced ability of the cyclic nucleotides to activate their specific kinases [147].

All these observations indicate the occurrence of a multistep resistance to antiaggregation in obesity and in obese type 2 diabetes mellitus, including the ability of insulin to increase NO, the ability of NO to increase cGMP, the ability of cGMP to reduce platelet calcium and consequently aggregation, and, similarly, the ability of PGI_2_ to increase cAMP and the cAMP ability to reduce platelet function [34, 141]. Many of these alterations described in obese subjects are reverted by weight loss [148].

## 5. Adipokines, Cytokines, and Hemostatic Balance

Omental adipose tissue is a dynamic endocrine organ which secretes a number of bioactive peptides involved in the control of insulin action, energy homeostasis, inflammation, and cell growth by autocrine, paracrine, and endocrine actions [149, 150]. Some of these factors, indicated as adipokines, are directly synthesized by adipocytes [150]; others are produced and released from inflammatory cells as a consequence of the presence of hypertrophied adipocytes with altered adipokine synthesis profile which trigger an increased number of macrophages through the production of chemokines, such as monocyte chemoattractant protein-1 (MCP-1) [151].

Adipokines locally regulate fat mass by modulating adipocyte size/number or angiogenesis [150, 152, 153]; furthermore, as endocrine mediators, they are involved in the control of appetite and energy balance, immunity, insulin sensitivity, angiogenesis, blood pressure, lipid metabolism, and hemostasis [153]. Evidence indicates that some adipokines play a role also in the cardiovascular disease linked to central obesity [149, 150].

Cytokines from monocytes and macrophages, such as TNF*α*, IL6, and MCP-1 itself, which contribute to the local and systemic proinflammatory state are mainly represented [154].

Increased fat mass leads to dysregulation of adipose tissue activity with oversecretion of deleterious adipokines and hyposecretion of beneficial ones (adiponectin, in particular) [155, 156].

In this part of the paper some relevant data concerning several adipokines and cytokines recognized as involved in the derangement of the systemic hemostatic balance in patients with central obesity will be considered: in particular, leptin, ghrelin, adiponectin, and inflammatory cytokines considering their role in hemostatic balance, platelet function, and coagulative alterations characterizing central obesity.

### 5.1. Adipokines

Adipokines are circulating molecules with a central role in the pathophysiology of obesity and its systemic health effects [153]; in addition, they modulate the production of inflammatory mediators [153].

#### 5.1.1. Leptin

Leptin is a 167-amino acid adipokine mainly produced by mature adipocytes, which primarily regulates food intake and energy expenditure [151]. Both human and animal studies showed that its circulating levels are directly related to adipose tissue mass, presumably to inform the brain regarding the quantity of stored fat [151]; however, in humans, increased leptin levels do not cause weight loss due to selective resistance to leptin metabolic actions [157, 158].

Leptin influences also angiogenesis, inflammation, arterial pressure, and secretion of other adipokines; in humans, the vascular actions of leptin are considered proatherogenic, and increased circulating levels of leptin are recognized as an independent risk factor for cardiovascular diseases [158–162] and cerebrovascular events [163, 164].

At present, evidence indicates that plasma leptin concentrations are independently associated with the intima-media thickness of the common carotid artery [163, 164] and with the degree of coronary artery calcification in patients with type 2 diabetes mellitus, after controlling for adiposity and C-reactive protein [165, 166]; finally, hyperleptinemia could be involved in the increased risk of postangioplasty restenosis [167, 168]. 

The prothrombotic actions of leptin are related to an influence on platelet function and coagulation/fibrinolysis balance, resulting in enhanced agonist-induced platelet aggregation and increased stability of arterial thrombi [169–171]. 

Studies *in vitro* evidenced that leptin synergizes with subthreshold concentrations of agonists—such as ADP and thrombin—to induce platelet aggregation [169, 170, 172, 173]. Other actions on hemostatic balance contribute to induce a prothrombotic tendency [171]: in particular, circulating leptin levels positively correlate with a cluster of prothrombotic conditions, such as increased concentrations of PAI-1 [173–175], fibrinogen [176], vWF [176], and FVIIa [177], and negatively correlate with protective factors such as tPA [163, 178], tissue factor pathway inhibitor [179], and protein C [179]. In addition, recent findings identified leptin as an inducer of functional TF in human primary neutrophils, suggesting its direct involvement in the coagulation mechanism by modulation of the extrinsic coagulation cascade [60, 61]. 

Finally, other mechanisms—such as inflammation, oxidative stress, endothelial dysfunction, and increased sympathetic tone—may contribute to leptin-induced vascular damage [168, 180–182].

#### 5.1.2. Adiponectin and Ghrelin

At present, the role of other adipokines in thrombosis is not fully defined [182]. Recent studies focused the attention on the effects of two adipocyte-derived hormones: adiponectin (an insulin-sensitizing hormone inversely correlated with adipose tissue mass) [91] and ghrelin (a growth hormone-releasing peptide exerting also orexigenic effects). Both adipokines exert protective effects against atherosclerosis by improving insulin resistance, dyslipidemia, and endothelial function [183–185]. Receptors for both adiponectin and ghrelin are present in circulating platelets [183]; however, *in vitro* studies failed to show that these adipokines modify platelet activation [181, 185].

The reduced levels of adiponectin, as well as the increased circulating concentrations of TNF-*α* and IL-6, may exert a prothrombotic role through enhanced adhesive molecule expression from endothelial cells and/or decreased NO bioavailability [183, 186–188].

#### 5.1.3. Resistin

Recent evidence showed that resistin, an adipokine involved in insulin resistance [91], exerts some vascular effects by inducing the expression of endothelial adhesion molecules such as VCAM-1 and ICAM-1, the endothelial production of endothelin-1, and the expression of CD40L [189]; furthermore, an association among resistin levels and markers of inflammation and fibrinolysis has been found [190] 

#### 5.1.4. Angiotensinogen

The renin angiotensin system is completely expressed in human adipose tissue, and the synthesis by adipocytes of angiotensinogen—precursor of the major vasoconstrictor hormone angiotensin II—is increased in central obesity [191–193]. 

Augmented angiotensinogen production by adipose tissue has been directly linked to angiogenesis and new adipose cell formation [194]; furthermore, the enhanced formation of angiotensin II stimulates the production of PAI-1 via the angiotensin II type 1 receptor [195], and the expression of adhesion molecule (ICAM-1, VCAM-1), MCP-1, and macrophage colony-stimulating factor (M-CSF) in vascular cells [196]. Angiotensin II also promotes the formation of ROS, thereby enhancing the oxidative stress and decreasing the availability of NO and increasing the risk of vascular tissue damage [197]. 

### 5.2. Inflammatory Cytokines

There is evidence that several alterations of hemocoagulative cascade and fibrinolysis in central obesity are closely related to the presence of low-grade inflammation [198].

#### 5.2.1. Tumor Necrosis Factor-*α* (TNF-*α*)

TNF-*α*, which is one of the inflammatory cytokines increased in obese subjects, is synthesized both by adipocytes and stromovascular cells; adipocytes, *per se*, predominantly produce unsecreted, membrane-bound TNF-*α*, which can act in an autocrine and paracrine fashion. The expression of TNF-*α* mRNA in adipose tissue correlates with body mass index as well as with percentage of body fat. 

The contribution of TNF-*α* to vascular impairment of central obesity is complex and still controversial [199]. Circulating TNF-*α* is involved in general systemic inflammation, which, in turn, impacts on the vasculature; furthermore, it impairs hemostatic balance by stimulating PAI-1 synthesis in human adipocytes [200].

#### 5.2.2. Transforming Growth Factor-*β* (TGF-*β*) and Thrombospondin-1 (TSP1)

TGF-*β* is a multifunctional cytokine with a relevant role in influencing the hemostatic balance; in particular, it stimulates PAI-1 biosynthesis in a large variety of cell types and increases circulating PAI-1 levels [106, 201], as evidenced in Figure 3. The relation with obesity has been showed by evidencing that the levels of TGF-*β* mRNA are significantly higher in the adipose tissue and that this cytokine may upregulate PAI-1 production in adipose tissue [106, 202]. Furthermore, the TGF-*β* and TNF-*α* pathways are interrelated, and these cytokines may cooperate to increase PAI-1 production.

 Finally, TGF-*β* acts as prothrombotic factor also by stimulating adipocytes to synthesize TF [58], as previously mentioned.

TSP1 is a multifunctional protein firstly isolated from platelets and megakaryocytes [203] and later also from adipocytes [204]; due to its prevailing expression in adipocytes compared with the stromal vascular fraction of adipose tissue, TSP1 is considered an adipokine [204].

 Beyond its inhibitory action on angiogenesis, TSP1 regulates cell proliferation, inflammation, and wound healing [205]; furthermore, it is a major regulator of TGF-*β* activity due to its ability to convert latent TGF-*β* procytokine to its biologically active form [206, 207].

TSP1 expression is increased in obese, insulin-resistant subjects and is positively associated with plasma PAI-1 levels, being one of the regulators of PAI-1 synthesis in adipose tissue [208].

## 6. Role of Atherogenic Dyslipidemia in the Impairment of Hemostatic Balance

Abnormal concentrations of lipids and apolipoproteins resulting from changes in the synthesis and catabolism of lipoprotein particles are typically observed in the patients with metabolic syndrome [209]. This mixed atherogenic dislipidemia is characterized by hypertriglyceridemia, low HDL cholesterol, predominance of small, dense LDL (sdLDL) particles, and accumulation of cholesterol-rich remnant particles (e.g., high levels of apolipoprotein B) [209]. sdLDL particles are atherogenic, being more prone to oxidation [210].

Circulating lipoprotein particles interplay with hemostatic factors modifying their expression and with platelets influencing their activation: therefore, mixed dislipidemia characterizing the metabolic syndrome plays a role in the prothrombotic tendency [211]. 

The reduction of circulating HDL particles, which are known to exert an antithrombotic activity by decreasing TF expression [212], may favour the activation of extrinsic coagulation pathway and upregulate thrombin generation. Furthermore, as previously mentioned, activation of FVII is closely related to the plasma levels of triglyceride-rich lipoproteins [65].

The increased plasma levels of VLDL particles, triglycerides, and free fatty acids and the decrease of circulating HDL particles may enhance the responses of circulating platelets [213]. 

## 7. Conclusions

Central obesity has a relevant impact on the risk of cardiovascular morbidity and mortality due to atherothrombotic events [6, 8, 9]. 

Together with hemodynamic alterations and metabolic impairment, central obesity accelerates the atherosclerotic vascular damage mainly through the presence of prothrombotic tendency and chronic low-grade systemic inflammation [29, 30].

As extensively reviewed, the prothrombotic tendency in central obesity is the result of a cluster of alterations involving intrinsic and extrinsic coagulation pathways, fibrinolysis and platelet function, each of which cooperates to favour the thrombotic processes [7, 10, 38, 106, 214]; furthermore, the increased number of circulating MPs—which act as a prothrombotic factor, delivering TF and other proteins which participate to the hemostasis mechanisms [44, 45, 71, 215]—is clearly involved in the coagulatory dysfunction of the metabolic syndrome [62, 75] and in the enhanced atherothrombotic risk [42, 72].

Growing evidence indicates that adipose tissue of the trunk and/or abdomen has a strong impact on vascular complications, through the production of mediators with paracrine and endocrine actions (adipokines and cytokines) [10, 106, 149, 150, 193, 216].

As discussed in the second part of this paper, changes in the synthesis and/or release of biologically active molecules as well metabolic alterations have to be considered as fundamental mechanisms involved in the adverse effects of adiposity on the vessel wall and hemostatic balance [10, 106, 149, 150, 195, 216].

Several mediators synthesized and released by adipose tissue in increased amount cause adverse effects through different mechanisms including the determination of a low-grade inflammatory milieu and oxidative stress; the production of other bioactive molecules with protective vascular effects is downregulated by the increase of the fat mass [188, 216, 217]. The alteration of hormonal pattern secreted by adipose tissue is involved in the alterations of the hemostatic balance, as well as in endothelial dysfunction [52, 111, 218], and in activation of circulating leukocytes [60, 61].

Due to the increasing impact of the central obesity across all ages, including children and adolescents, the prevalence of the metabolic syndrome has reached epidemic proportion both in western and developing countries. 

For the high risk of cardiovascular morbidity and mortality, metabolic syndrome represents a relevant problem in the clinical practice which requires an adequate definition of the complex cluster of pathogenetic factors [7, 10, 219], in order to propose multiple intervention strategies and to provide standardized guidelines, as reviewed in [220].

## Abbreviations

*α*2-AP: *α*2-antiplasmin
ADP: Adenosine 5-diphosphate
ARIC: Atherosclerosis risk in communities
BMI: Body mass index
cAMP: 3′,5′-cyclic adenosine monophosphate
CT: Computed tomography
cGMP: 3′,5′-cyclic guanosine monophosphate
ecNOS: Endothelial-type constitutive NO synthase
Ets: Endothelins
FDP: Fibrin degradation products
FVII: Factor VII
FVII:c: FVII coagulant activity
FVIII: Factor VIII
FIX: Factor IX
FX: Factor X
GTN: Glyceryl trinitrate
ICAM-1: Intercellular adhesion molecule-1
IL-6: Interleukin-6
LDL: Low-density lipoproteins
MCP-1: Monocyte chemoattractant protein-1
MRI: Magnetic resonance imaging
M-CSF: Macrophage colony-stimulating factor
MPs: Microparticles
NHANES III: National Health and Nutrition Examination Survey III
NCEP ATPIII: National Cholesterol Education Program Adult Treatment Panel III
NO: Nitric oxide
O_2_^−^: Superoxide anion
PAF: Platelet-activating factor
PAI-1: Plasminogen activator inhibitor-1
PAI-2: Plasminogen activator inhibitor-2
NPHS: Northwick Park Heart Study
PGI2: Prostacyclin
PMP: Platelet-derived microparticles
ROS: Reactive oxygen species
SNP: Sodium nitroprusside
sCD40L: Soluble CD40 ligand
sdLDL: Small, dense LDL
TAFI: Thrombin-activatable fibrinolysis inhibitor
TF: Tissue factor
t-PA: Tissue-type plasminogen activator
TGF-*β*: Transforming growth factor-*β*
TNF-*α*: Tumor necrosis factor-*α*
TSP1: Thrombospondin-1
TXA_2_: Thromboxane A_2_
u-PA: Urokinase-type plasminogen activator
VLDL: Verylow-density lipoproteins
VCAM-1: Vascular cell adhesion molecule-1
vWF: Von Willebrand factor
VSMC: Vascular smooth muscle cells
WHR: Waist-to-hip ratio
WHO: World Health Organization.
